# Study on Chemical Mechanical Polishing of Single-Crystal Diamond with a Novel Nicotinic Acid–Hydrogen Peroxide Green Slurry

**DOI:** 10.3390/mi17070833

**Published:** 2026-07-13

**Authors:** Jixiang Yi, Longxing Liao, Yiming Fang

**Affiliations:** 1 College of Marine Equipment and Mechanical Engineering, Jimei University, Xiamen 361021, China; 2 College of Mechanical and Automotive Engineering, Xiamen University of Technology, Xiamen 361024, China

**Keywords:** single-crystal diamond, slurry, CMP

## Abstract

Single-crystal diamond (SCD) has the characteristics of a hard surface and stable chemical properties, making it difficult to achieve ultra-smooth and ultra-low-damage surface polishing using conventional polishing slurries. In this study, a novel green chemical mechanical polishing (CMP) slurry containing only hydrogen peroxide, nicotinic acid, silica (SiO_2_) abrasive particles and deionized water was developed to achieve ultra-smooth, ultra-low-damage (0.5 nm) and atomic-scale surface roughness (Ra 0.473 ± 0.035 nm) polishing of SCD. Additionally, the influence of diamond, silicon carbide and SiO_2_ abrasive particles on the surface quality of SCD after CMP was investigated by single-factor experiments. Based on XPS characterization, the mechanism of SCD CMP was revealed: the SCD surface was first oxidized to form C-O and C=O groups, and then these groups were removed under the mechanical action of SiO_2_ abrasives, ultimately achieving atomic-scale removal of the material.

## 1. Introduction

Single-crystal diamond (SCD) exhibits exceptional physical, chemical, optical, and thermal properties, including high hardness, a low friction coefficient, and outstanding thermal conductivity. It finds extensive applications in various fields, such as semiconductor devices, cutting tools, and optical windows [1,2,3,4]. Chemical vapor deposition and high-pressure, high-temperature methods are the primary techniques employed for synthesizing diamonds. These approaches overcome the limitations of the scarcity, small size, and high price of natural diamonds while expanding the range of diamond applications [5]. However, synthetic diamonds often exhibit defects such as large grain sizes and rough surfaces that hinder their ability to meet the requirements for high-performance diamond devices [6,7]. Therefore, research on techniques for achieving high-quality diamond surfaces holds significant importance.

The main methods for achieving high-quality diamond surfaces include mechanical polishing, plasma-assisted polishing and chemical mechanical polishing (CMP) [3,4,8,9,10]. Mechanical polishing involves the high-speed grinding of the diamond surface using specialized grinding wheels. Although it has high polishing efficiency, the resulting surface often has scratches, a large damage layer (>2 nm), and a rough surface (Ra > 1 nm) [11,12]. For example, Zhou et al. [13] developed a chromium-coated diamond grinding wheel for rapid machining of diamond films, which resulted in a surface roughness of 35.8 nm after polishing. Lu et al. [14] designed a novel Sol–Gel polishing pad for high-speed polishing of SCD that reduced the surface roughness from Ra 78.024 nm to 6.126 nm within 2 h.

In recent years, numerous scholars have discovered that plasma-assisted polishing technology can achieve damage-free polishing of SCD and attain surface quality at the atomic scale. For example, Luo et al. [15] conducted a study on the polishing process of SCD using atmospheric-pressure inductively coupled plasma, achieving a damage-free and ultra-smooth surface. Within 120 min, the surface roughness decreased from Sa 308 nm to 0.86 nm. Liu et al. [16] employed nanosecond pulse laser conditioning combined with plasma-assisted polishing to polish SCD substrates, obtaining atomically smooth surfaces (Ra 0.51 nm) without inducing any damage. However, plasma-assisted polishing exhibits low efficiency and incurs high equipment costs, thereby limiting its widespread utilization.

Compared to alternative methods, CMP offers the advantage of simultaneously achieving high efficiency and quality while maintaining low equipment costs and being a relatively simple process. CMP utilizes the synergistic effect of chemical corrosion and mechanical removal to effectively polish workpieces [17]. For instance, Thomas et al. [17] utilized Syton colloidal silica alkaline polishing slurry (15–50 wt% SiO_2_, pH = 9.2–10.1, 4–5 wt% ethylene glycol) to polish single-crystal diamond. However, the ethylene glycol used in this slurry is a long-chain organic compound with poor natural biodegradability. Lin et al. [18] utilized 10% KNO_3_ as an oxidant along with 3 μm diamond particles for polishing SCD, resulting in an Ra value of approximately 1.0 nm with a damage layer thickness of about 2.3 nm. Liao et al. [19,20] investigated the impact of different oxidants and pH values on the performance of SCD polishing, developing a novel slurry comprising silica, ferrous sulfate, hydrogen peroxide, nitrilotriacetic acid, and deionized water for the polishing process. The achieved average Ra value of the SCD surface was 0.754 nm, with a damage layer thickness of 0.7 nm. Yuan et al. [21] developed a slurry containing Fe^3+^, H_2_O_2_, KOH, HCl and TiO_2_ for UV-assisted CMP of diamond, which resulted in amorphous carbon damage on the diamond surface with a damage layer thickness of 0.6 nm.

The literature above demonstrates that CMP can achieve low-damage and smooth surface polishing of SCD [22]. However, the slurry used typically consists of complex compositions, primarily containing metal ions and strong corrosive reagents, which are not environmentally or operator-friendly. Additionally, limited research has been conducted on the effects of different abrasive particles on the surface quality of SCD achieved by CMP. This paper develops a green and environmentally friendly slurry and investigates the influence of different abrasive particle materials on the SCD surface quality after CMP by using the single-factor test method. Moreover, the characterization techniques employed include optical microscopy, atomic force microscopy (AFM), and transmission electron microscopy (TEM) to analyze surface scratches, roughness, corrugation, and damaged layer thickness. Finally, the mechanism of SCD CMP was revealed through X-ray photoelectron spectroscopy (XPS) characterization.

## 2. Materials and Methods

**Figure 1:**
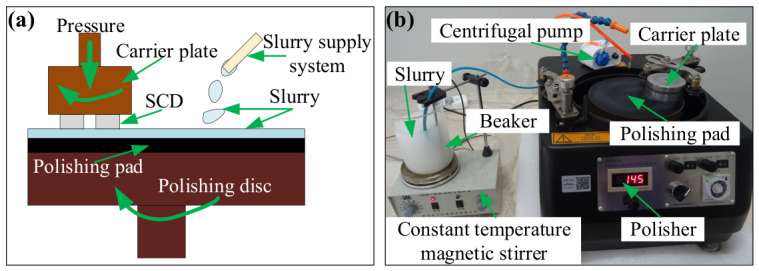
CMP of SCD: (**a**) Schematic diagram of the SCD CMP process and (**b**) photo of the CMP system.

Figure 1 shows the principle of SCD CMP and the practical polishing process, as well as the components of the CMP system, which include a polisher, centrifugal pump, constant-temperature magnetic stirrer, beaker, polishing slurry, carrier plate, and workpiece. The polisher used is the UNIPOL-802 automatic precision grinding and polishing machine (Shenyang Kejing, Shenyang, China), which has a 200 mm diameter polishing disk. The test employed SCD samples with dimensions of 8 × 8 × 0.25 mm^3^, which were developed and provided by the Ningbo Institute of Materials Science, Chinese Academy of Sciences. The samples were grown via chemical vapor deposition (CVD) without post-processing, and their raw surface roughness Ra was 6.490 ± 0.442 nm. Three SCD samples were uniformly bonded to the carrier plate using paraffin wax and were polished by relative rotation with the polishing disk under the assisted positioning action of the holder. The parameters for the polishing process were set to 145 r/min for the polishing disk, 268 kPa for the polishing pressure, 25 mL/min for the polishing slurry flow rate, and 3.5 h for the polishing time.

Three kinds of slurries with different abrasive particles—diamond, silicon carbide (SiC), and SiO_2_ powders—were developed. Then, SCD single-factor experiments were conducted sequentially; the specific experimental design is shown in Table 1. To develop the slurries, deionized water was added to the beakers, followed by 4 wt% of H_2_O_2_, 3 g/L of nicotinic acid (NA), and 5 g/L of abrasive particles. After preparing the slurry, it was subjected to ultrasonic action using a cleaning device for 30 min to fully dissolve the components. The beaker was then placed on the tray of the constant-temperature magnetic stirrer to ensure constant-temperature heating with magnetic stirring, maintaining the polishing slurry at a constant temperature of 25 °C and ensuring even mixing of each reagent component with the abrasive particles to prevent precipitation. The slurry was transported by a centrifugal pump and dripped onto the polishing pad. The polishing pad was rotated by the polishing disk to evenly disperse the slurry on the pad and allow it to come into contact with the SCD surface for CMP.

The H_2_O_2_ solution had a concentration of 30%. The molecular mass of NA was 123.11, and its purity was 99%. The abrasive particles were all powder particles with a size of 50 nm. Diamond particles had a Mohs hardness of 10, while SiC particles had a Mohs hardness of 9.5, and SiO_2_ particles had a Mohs hardness of 5.5–6.5.

The slurry contained only two chemical reagents: H_2_O_2_ and NA. Both are simple in composition. H_2_O_2_ decomposes into water and oxygen, which is environmentally friendly. NA, also known as vitamin B3, has low toxicity to humans, making it fundamentally different from the strong acidic, alkaline and highly corrosive additives used in conventional polishing slurries. It will not cause corrosive irritation to the skin or respiratory tract of operators during slurry preparation and polishing operations. In addition, nicotinic acid is biodegradable. After wastewater discharge, it will not accumulate in the environment over the long term, thus eliminating persistent ecological hazards [23,24]. These characteristics make the slurry environmentally friendly and safe for operators.

**Figure 2:**
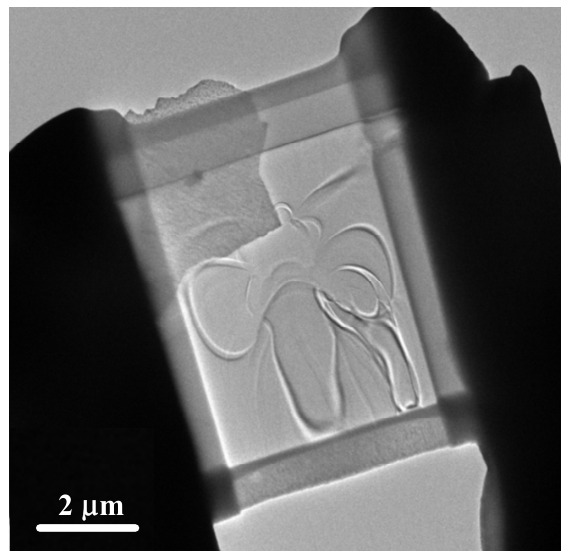
Cross-sectional TEM specimen.

The optical morphology of the SCD sample surface was characterized using an Olympus metallurgical microscope (MX40, Tokyo, Japan) with a scale of 200 μm. The SCD surface roughness was measured before and after polishing using AFM (NanoWizard UItra Speed &inVia Raman, Berlin, Germany) with a detection range of 10 × 10 μm^2^, and three distinct regions were measured for each sample. A dual-beam FIB scanning electron microscope (Helios-G4-CX, Hillsboro, OR, USA) was used to prepare TEM cross-section samples of SCD. The cutting technique was employed, and the resulting cross-section, shown in Figure 2, extended from the polished surface to the sample substrate and had a thickness of approximately 3 μm. To prevent the ion beam from damaging the sample surface during preparation, a layer of silver adhesive was applied, followed by a protective Pt layer. The cross-section samples were analyzed using TEM (Talos F200x, Hillsboro, OR, USA) observation. The surfaces of the SCD samples were characterized using XPS (Thermo Scientific K-Alpha, East Grinstead, UK) before polishing, after immersion in the slurry for 12 h, and after polishing. The XPS system employed an Al Kα radiation source (1486.6 eV), operating at an anode voltage of 12 kV and emission current of 6 mA. The measurement data was calibrated using the C1s peak at 284.8 eV and fitted utilizing Avantage (v5.948) software. An electronic balance (ME204E, Greifensee, Switzerland) with a resolution of 0.1 mg was adopted to measure the variation in sample mass before and after polishing. Each sample was measured three times, and the material removal rate was calculated via Equation (1):

(1) $$MRR(\mathrm{nm}/\min)=\frac{\Delta m}{\rho \cdot s\cdot t}\times 10^{6}$$

where Δ*m* is the difference in the mass of the workpiece before and after polishing (mg); *ρ* represents the density of the workpiece (g/cm^3^); *t* denotes the polishing time (min); and *s* is the polished surface area of the workpiece (mm^2^).

## 3. Results

**Figure 3:**
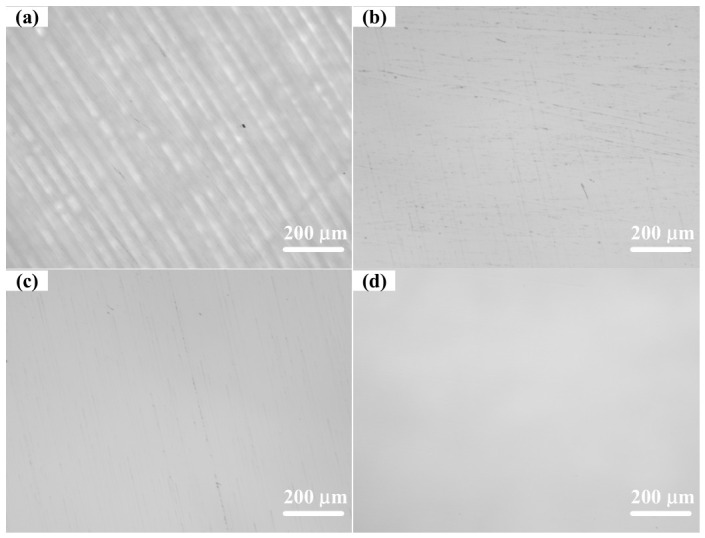
Optical photographs of SCD: (**a**) raw surface and surfaces after CMP with slurries containing (**b**) diamond, (**c**) SiC, and (**d**) SiO_2_.

Figure 3 displays an optical photograph of the SCD surface. It can be observed that the original, raw surface of SCD is very rough, with wide grooves (a). However, after polishing with the slurry containing diamond abrasive particles, the grooves become thinner, leaving behind dense, fine scratches (b). Polishing with the slurry containing SiC abrasive particles results in a relatively flat and smooth surface, but noticeable polishing scratches remain (c). Finally, polishing with the slurry containing SiO_2_ abrasive particles results in a smooth surface without any scratches (d).

**Figure 4:**
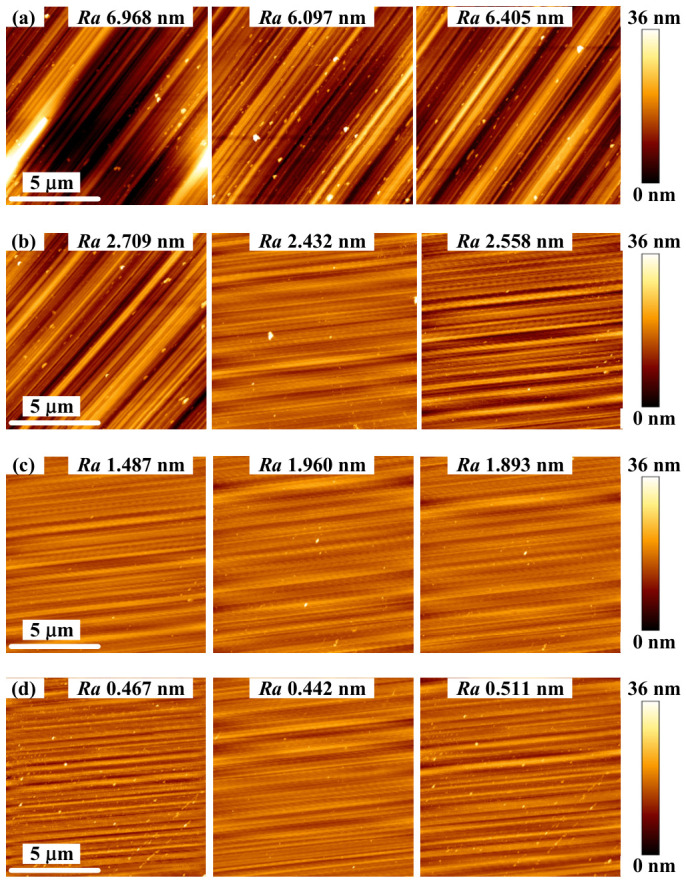
2D AFM photographs of different regions of SCD: (**a**) raw surface and surfaces after CMP with slurries containing (**b**) diamond, (**c**) SiC, and (**d**) SiO_2_.

**Figure 5:**
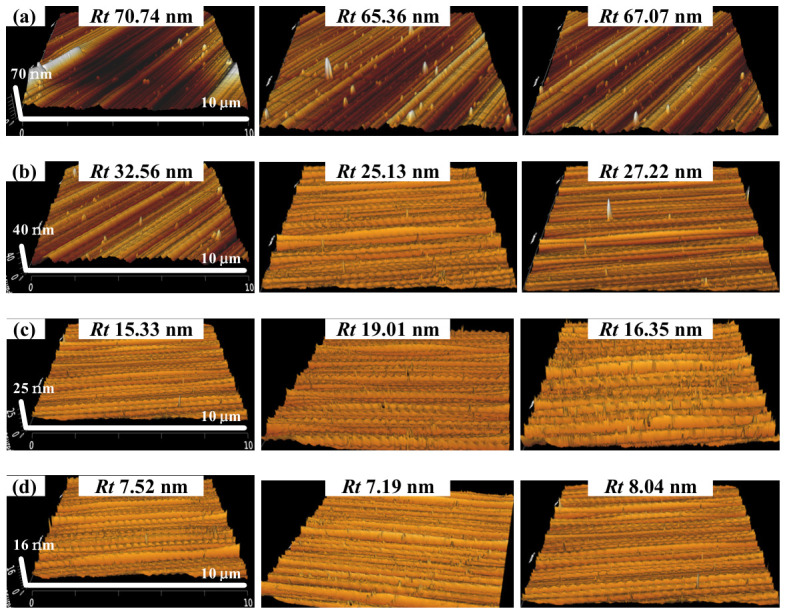
3D AFM photographs of different regions of the SCD: (**a**) raw surface and surfaces after CMP with slurries containing (**b**) diamond, (**c**) SiC, and (**d**) SiO_2_.

Figure 4 and Figure 5 present the 2D and 3D morphologies of the SCD surface, respectively. Based on the results of AFM characterization, it is evident that the raw SCD surface exhibits a high level of roughness (Ra 6.490 ± 0.442 nm), displaying significant corrugation (Rt 67.723 ± 2.749 nm). After polishing with the diamond abrasive slurry, the surface grooves become smaller, and the protruding uneven ripples are relatively reduced (Rt 28.303 ± 3.832 nm), resulting in an overall improvement in surface roughness (Ra 2.566 ± 0.139 nm), as shown in Figure 4b and Figure 5b. When utilizing the SiC abrasive slurry for polishing, the prominent ripples on the SCD surface significantly decrease (Rt 16.897 ± 1.900 nm), leading to a comparatively smoother overall texture (Ra 1.780 ± 0.256 nm), but noticeable grooves still persist (Figure 4b and Figure 5b). Finally, by employing the SiO_2_ abrasive slurry for polishing, there is a remarkable reduction in ripple formation on the SCD surface (Rt 7.583 ± 0.429 nm), resulting in a fine and smooth texture with exceptional quality (Ra 0.473 ± 0.035 nm).

Based on the analysis illustrated in Figure 3, Figure 4 and Figure 5, the developed green slurry can effectively improve the surface quality of SCD, but the polishing effect is different when the slurry contains different abrasive particles. The results indicate that SiO_2_ abrasive particles have the most significant polishing effect, followed by SiC abrasive particles. Diamond abrasive particles, on the other hand, have the least improvement effect on surface roughness after polishing. Possible reasons for the observed phenomena include the hardness of the diamond particles, strong mechanical friction on the SCD surface, the ease of scratch production, and poor polishing effects. Meanwhile, diamond abrasives achieve the maximum MRR of 49.46 ± 1.32 nm/h. Although the hardness of SiC particles (9.5) is lower than that of diamond particles, it is still high and can cause friction on the polishing sample, leading to scratches on the SCD surface and resulting in subpar polishing quality, with an MRR of 43.71 ± 1.01 nm/h. On the other hand, the hardness of SiO_2_ particles is relatively low, causing minimal scratches on the SCD and reducing the likelihood of damage to its surface. As a result, the quality of the polishing is much better, while its MRR is relatively low at 40.88 ± 0.56 nm/h.

**Figure 6:**
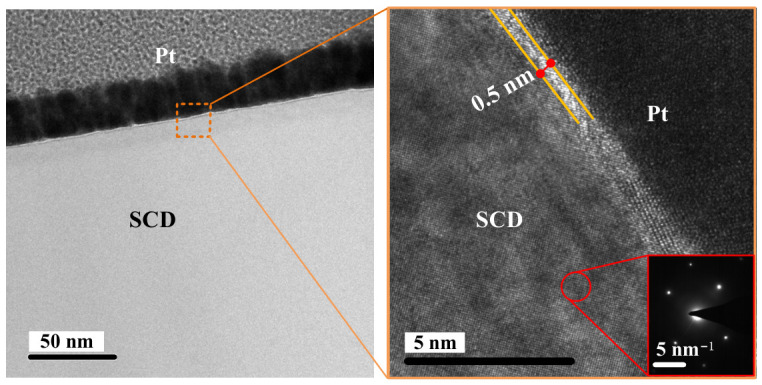
TEM photograph of SCD surface after CMP with slurry containing SiO_2_ abrasive particles.

Figure 6 characterizes the TEM photographs of the SCD surface after polishing with the slurry containing SiO_2_ abrasive particles. The results of the characterization indicate that the polished SCD surface is smooth and scratch-free. Additionally, the thickness of the surface damage layer is only 0.5 nm. These findings further support the use of green slurry containing H_2_O_2_, NA, SiO_2_ and deionized water for SCD CMP to achieve an ultra-smooth and ultra-low-damage polished surface of SCD.

**Figure 7:**
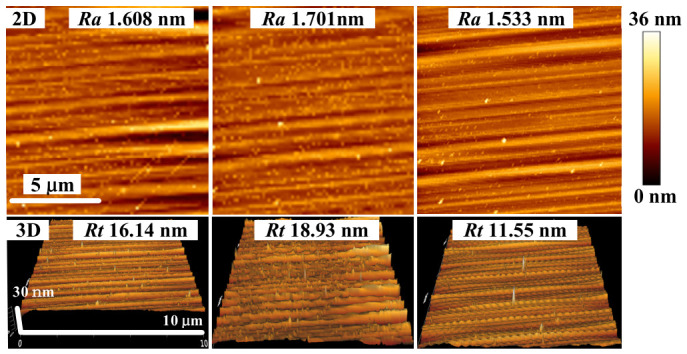
2D and 3D AFM photographs of different regions of SCD.

To investigate the role of NA during polishing, a control experiment without NA, using slurry consisting of only SiO_2_ and H_2_O_2_, was designed. Figure 7 presents the 2D and 3D AFM characterization results of the SCD surface obtained under this condition, with a surface roughness of Ra = 1.164 ± 0.084 nm and Rt = 15.540 ± 3.726 nm, as well as an MRR of 35.62 ± 0.70 nm/h. Compared with Test 3, in which the slurry contained H_2_O_2_, SiO_2_ and NA simultaneously, the specimen without NA exhibits larger surface roughness and a lower MRR. This demonstrates that the addition of NA contributes to improving the surface quality and material removal rate of polished SCD substrates.

Table 2 summarizes the Ra, Rt and MRR values under different polishing slurry compositions.

**Figure 8:**
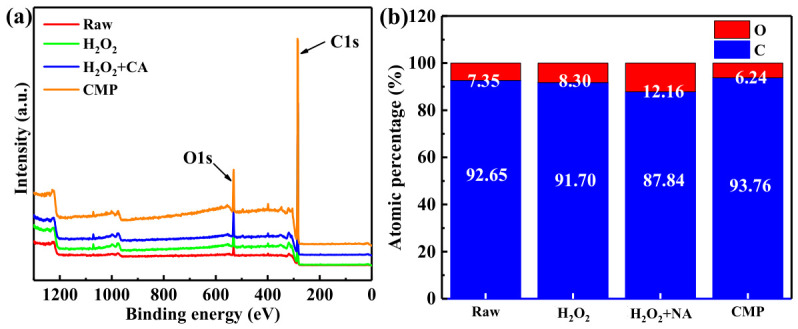
XPS measurements of SCD: (**a**) XPS spectra of the SCD surface and (**b**) the atomic percentages of C and O elements on the surface.

Figure 8 displays the full XPS spectra of the SCD surface before polishing, after immersion in H_2_O_2_, after immersion in H_2_O_2_ + NA, and after polishing, along with the atomic percentages of C and O elements. The results in Figure 8a indicate clear wave peaks at binding energies of 284.8 and 531.5 eV, corresponding to C 1s and O 1s, respectively, indicating no change in elemental composition. On the other hand, Figure 8b illustrates the atomic percentages of C and O elements and suggests that the O element on the SCD surface increases significantly from 7.35% to 8.30% after immersion in pure H_2_O_2_. In contrast, immersion in the H_2_O_2_ + NA mixture leads to a remarkable increase in oxygen content from the initial 7.35% to 12.16%, which subsequently drops to 6.24% after CMP. This indicates that new oxygen-containing groups formed on the SCD surface when it was immersed in the polishing slurry, and these were then removed by mechanical action during the CMP process. Furthermore, NA facilitates the formation of such new oxygen-containing groups.

**Figure 9:**
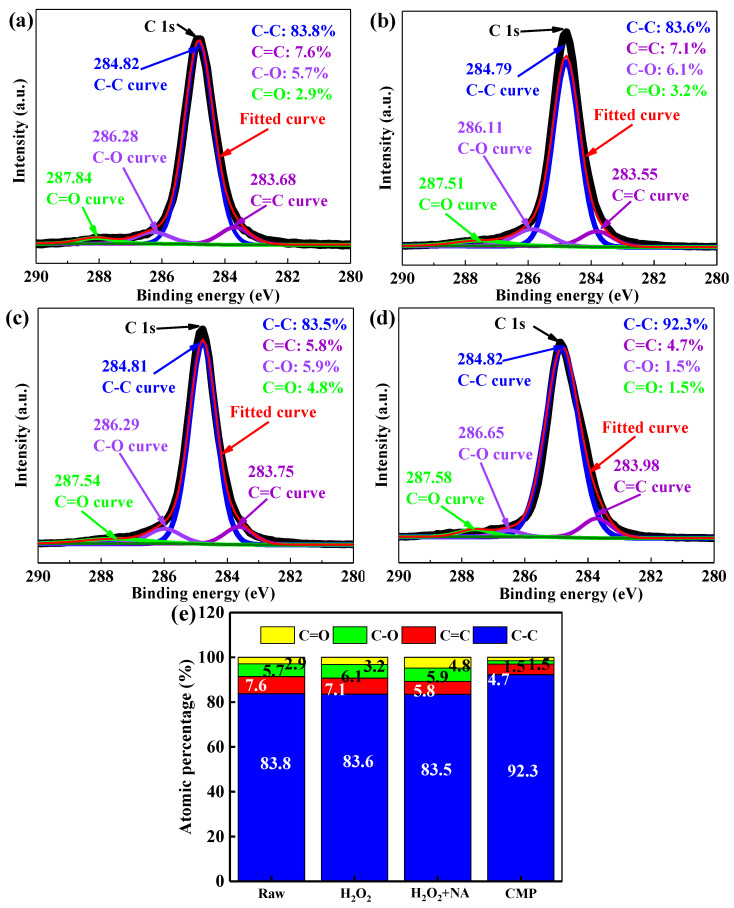
High-resolution XPS survey spectra of C 1s on SCD surfaces: (**a**) before polishing, (**b**) after H_2_O_2_ immersion, (**c**) after H_2_O_2_ + NA immersion, and (**d**) after polishing, and (**e**) the atomic percentage of each group.

Figure 9 presents the high-resolution XPS survey spectra of C 1s on the SCD surfaces before polishing, after immersion in H_2_O_2_, after immersion in H_2_O_2_ + NA, and after polishing, along with the atomic percentage of each group. Based on the existing literature [25], the peaks of binding energy at 283–284, 284–285, 286–287, and 287–288 eV correspond to C=C, C-C, C-O, and C=O groups, respectively.

Figure 9 illustrates that the form of C elements on the surface of SCD remains constant, specifically the C-C groups corresponding to the peak of binding energy at 284.8 eV. However, the proportion of each group varies under different conditions (Figure 9e). Comparing Figure 9a with Figure 9b, it can be observed that the percentage of C=C groups decreased from 7.6% to 7.1% after SCD was immersed in pure H_2_O_2_ solution; meanwhile, the percentages of C–O and C=O groups rose from 5.7% and 2.9% to 6.1% and 3.2%, respectively. Furthermore, comparing Figure 9b with Figure 9c reveals that the proportion of C=C groups declined further, accompanied by a remarkable increase in the C=O group content, upon immersion in the mixed H_2_O_2_ + NA solution. By contrast, comparing Figure 9c with Figure 9d indicates that the percentages of C–O and C=O groups dropped sharply from 5.9% and 4.8% to 1.5% and 1.5%, showing an obvious reduction in their contents. The findings further indicate that oxidation reactions take place between H_2_O_2_ and carbon atoms on the SCD surface under the chemical effect of the slurry to generate C–O and C=O functional groups, and NA can promote this oxidation reaction. These groups are then removed during the CMP process by the mechanical action of the SiO_2_ abrasive particles, achieving atomic-scale material removal.

## 4. Conclusions

A novel green slurry of SCD CMP was developed in this study. The influence of different abrasive particles on the polishing quality of the SCD surface was investigated, and the mechanism of SCD CMP was revealed. The relevant conclusions are as follows:

(1) The slurry containing only H_2_O_2_, NA, SiO_2_ and deionized water could achieve ultra-smooth, ultra-low-damage and atomic-scale surface roughness polishing of SCD.
(2) The SCD surface obtained by the diamond abrasive particles slurry has more scratches, and the formed surface quality is poor. Similarly, the surface polished with the SiC abrasive particles slurry also has noticeable scratches and average surface quality. However, the SCD surface polished with the SiO_2_ slurry is smooth and scratch-free and has a small damage layer, resulting in a high-quality surface.
(3) The mechanism of SCD CMP is that the slurry oxidizes the C atoms on the SCD surface, forming C-O and C=O groups, and then mechanically removes the groups, ultimately achieving the atomic-scale removal of C atoms.

Although high-quality SCD surfaces were obtained by exploring polishing mechanisms, this work has several limitations requiring further study:

(1) Single-variable tests only adjusted abrasives with fixed nicotinic acid-containing slurry. No blank groups without nicotinic acid were tested to verify its independent role, nor were oxidants and inhibitors systematically studied.
(2) The dynamic balance between chemical and mechanical effects remains unclear.
(3) Only surface roughness was characterized; the material removal rate, reflecting polishing efficiency, was not measured.

## Figures and Tables

**Table 1:** Single-factor experimental design.

Test Number	Abrasive Particles	Slurry Composition	Process Parameters
1	Diamond	50 nm 5 g/L abrasive particles, 4 wt% H_2_O_2_ solution, 3 g/L NA	n = 145 rpm, *p* = 268 kPa, f = 25 mL/min, t = 3.5 h
2	SiC		
3	SiO_2_		

**Table 2:** Surface roughness and MRR corresponding to different slurry compositions.

Slurry Composition	Ra (nm)	Rt (nm)	MRR (nm/h)
Diamond, H_2_O_2_, NA	2.566 ± 0.139	28.303 ± 3.832	49.46 ± 1.32
SiC, H_2_O_2_, NA	1.780 ± 0.256	16.897 ± 1.900	43.71 ± 1.01
SiO_2_, H_2_O_2_, NA	0.473 ± 0.035	7.583 ± 0.429	40.88 ± 0.56
SiO_2_, H_2_O_2_	1.614 ± 0.084	15.540 ± 3.726	35.62 ± 0.70

## Data Availability

The original contributions presented in this study are included in the article. Further inquiries can be directed to the corresponding author.
